# Comprehensive case report and literature review on perioperative management of multiple pheochromocytoma in a pediatric patient

**DOI:** 10.3389/fped.2025.1439186

**Published:** 2025-02-11

**Authors:** Shenghua Yu, Tianxiao Zou, Sisi Wei, Yani Yu, Guili Ding

**Affiliations:** ^1^ Department of Anesthesiology, Shanghai Children’s Hospital, School of Medicine, Shanghai Jiao Tong University, Shanghai, China; ^2^ Department of SICU, Shanghai Children’s Hospital, School of Medicine, Shanghai Jiao Tong University, Shanghai, China

**Keywords:** pheochromocytoma, paraganglioma, pediatric endocrinology, hypertension, perioperative management

## Abstract

Pheochromocytomas (PCCs) are rare neuroendocrine tumors originating from the adrenal medulla, particularly uncommon in pediatric populations. This case report presents a 12-year-old boy with a three-year history of fatigue and a one-year history of blurred vision, who was admitted with severe hypertension (230/200 mmHg). Abdominal CT imaging revealed bilateral adrenal masses measuring 40 mm on the left and 12 mm on the right. The diagnosis of pheochromocytoma was confirmed by elevated blood catecholamine metabolites. During perioperative preparation, the patient experienced complications, including polyuria, thrombosis, and infection, necessitating an emergency resection of the right adrenal tumor. This intervention led to a successful recovery. Subsequent genetic testing identified a mutation in the VHL gene. After further medical management, the left adrenal tumor was also successfully removed. At one-year follow-up, the patient remained normotensive with no signs of recurrence. This case emphasizes the critical role of genetic testing and cutious perioperative management in the treatment of pediatric pheochromocytoma.

## 1 Introduction

Pheochromocytomas (PPC) and paragangliomas (PGL) are neuroendocrine tumors originating from the adrenal medulla and/or extra-adrenal glands. Collectively known as PPGL, these tumors secrete large amounts of catecholamines such as dopamine, epinephrine, and norepinephrine, leading to various clinical symptoms and serious complications. PPGL is a rare endocrine tumor with an annual incidence of approximately 1 in 300,000, 20% of which are diagnosed in children and adolescents. PCC account for 80%–85% of these cases, while paragangliomas make up 15%. The prevalence of PPGL in hypertensive adults is 0.2%–0.6%, whereas in hypertensive pediatric patients, it is 1.7% (1). Approximately 10% of PCCSs are malignant and hereditary in adults. In children, 66% of PPGL cases are PGL, 24% are bilateral, and 80% have a family history or symptoms (2). Moreover, 50% of patients have metastases, 20% of which occur in children (3). The most common manifestations of PPGL include headaches, palpitations, and hyperhidrosis, characterized by paroxysmal hypertension and hypovolemia. Blood pressure control and fluid management are critical before surgery for most children.

## 2 Case description

A 9-year-old boy, weighing 20 kg, was admitted to the hospital due to fatigue for over 3 years and blurred vision for over 1 year. His highest recorded blood pressure was 230/200 mmHg, and his blood showed high levels of catecholamine metabolites. Abdominal CT revealed bilateral adrenal masses, with the left tumor measuring 40 mm and the right 12 mm. Based on medical history, blood tests, and imaging, he was diagnosed with pheochromocytoma. Various antihypertensive drugs, including betalocol, phentolamine, and phenoxybenzamine, were administered to manage blood pressure. During the perioperative period, the patient developed a series of complications, including fever, diarrhea, pulmonary infiltrates, and abnormal liver function, characterized by elevated liver enzymes (ALT 134 U/L, AST 233 U/L), hypoalbuminemia (22 g/L), and electrolyte imbalances (Na^+^ 129 mmol/L, K^+^ 3.2 mmol/L, Cl^−^ 90 mmol/L, Mg^2+^ 0.57 mmol/L). Coagulation abnormalities were also observed, with a prolonged prothrombin time (PT) of 25.8 s and activated partial thromboplastin time (aPTT) of 45.7 s. Subsequently, the patient experienced polyuria and a left femoral vein thrombosis, with the worsening polyuria further exacerbating the electrolyte disturbances. This situation prompting an emergency resection of the right adrenal tumor, which was pathologically confirmed as pheochromocytoma.

At age 12, weighing 44 kg, follow-up revealed enlargement of the left adrenal tumor, and genetic testing indicated a heterozygous *VHL* gene mutation, diagnosing him with von Hippel-Lindau syndrome. Phenoxybenzamine, amlodipine, and metoprolol were administered successively to decrease blood pressure, venous blood volume was supplemented before the operation, and the right adrenal tumor was resected under laparoscopy. One year post-surgery, his blood pressure stabilized near normal levels.

### 2.1 Diagnosis, treatment, and outcomes

During the initial hospitalization, blood tests revealed elevated levels of free methoxyepinephrine (1.0 pg/ml, reference range <62 pg/ml), methoxynorepinephrine (207.4 pg/ml, reference range <145 pg/ml), and vanillylmandelic acid (VMA) (29.6 mg/24 h, reference range <13.6 mg/24 h) in a 24 h urine sample. Abdominal CT imaging identified a mass measuring 20.8 × 41.2 × 40.9 mm in the right adrenal gland and a 12.3 mm mass in the left adrenal gland. Echocardiography showed aortic dilation, interventricular septal hypertrophy, and mild mitral regurgitation, indicating long-standing hypertension. The patient's blood pressure improved with treatment using phentolamine and phenoxybenzamine.

During hospitalization, a femoral vein puncture was performed, and an intravenous catheter was placed to meet clinical treatment needs. Subsequently, routine follow-up with Doppler ultrasound revealed left femoral vein thrombosis. The patient also developed polyuria and electrolyte imbalances, characterized by reduced urine osmolality and near-normal plasma osmolality. A multidisciplinary consultation concluded that the adrenal mass was likely causing a disturbance in water and salt metabolism. However, mineralocorticoid supplementation failed to resolve these symptoms. Preoperative vital signs showed blood pressure ranging from 120–150/75–100 mmHg, heart rate between 90 and 160 bpm, and respiratory rate of 20–32 breaths per minute. The patient also presented with liver and kidney function impairment, along with prolonged coagulation time. Due to the severity of the condition, an urgent resection of the right adrenal tumor was performed.

The highest blood pressure recorded during surgery was 165/111 mmHg. Phentolamine and esmolol were administered intermittently to manage blood pressure and heart rate. Blood pressure decreased to 103/84 mmHg after ligation of the tumor veins. The left adrenal gland appeared normal in size, with a palpable 10 mm mass that did not cause significant changes in heart rate or blood pressure when touched. Given the critical condition, only the right adrenal tumor was removed. The surgery lasted approximately 4 h, during which 1,600 ml of balanced salt solution, 150 ml of red blood cells, and 200 ml of plasma were administered. Blood loss was approximately 80 ml, and urine output was 1,500 ml. Pathological analysis, including immunohistochemical staining, confirmed the diagnosis of pheochromocytoma. The results were as follows: Ki-67: 3%–5%, Chromogranin A: Weakly positive, Neuron-Specific Enolase (NSE): Positive, Cytokeratin (CK): Negative, Epithelial Membrane Antigen (EMA): Negative. Postoperatively, the patient was prescribed warfarin to address femoral vein thrombosis, and 9-*α* hydrocortisone and hydrocortisone acetate to supplement glucocorticoid and mineralocorticoid levels. Two months later, urine VMA levels normalized (2.7 mg/24 h).

During postoperative follow-up, the left adrenal mass showed enlargement, while the patient's blood pressure stabilized at 110/64 mmHg. Preoperative blood tests revealed methoxyepinephrine at 20.02 nmol/L and methoxynorepinephrine at <0.08 nmol/L. Abdominal MRI showed postoperative changes in the right adrenal gland and a mass in the left adrenal gland measuring 39.2 mm × 28.0 mm × 68.4 mm. Genetic testing identified a heterozygous mutation in the VHL gene (c. 482G > A/p. Arg161Gln), confirming the diagnosis of von Hippel-Lindau syndrome. To manage blood pressure, the patient was treated with phenoxybenzamine (starting at 10 mg, increasing to 20 mg every 12 h), amlodipine (5 mg daily), and metoprolol (25 mg daily). Sodium intake was increased to restore blood volume over a period of approximately three weeks. During surgery, the patient's hemodynamics remained stable, with blood pressure ranging from 97–120/56–76 mmHg. The total fluid intake was approximately 6.0 ml/kg/h, including 150 ml of red blood cells, 20 g of albumin, and urine output of 2.25 ml/kg/h.

Post-discharge, the patient continued taking oral 9-*α*-hydrocortisone and hydrocortisone acetate to supplement glucocorticoids and salt corticosteroids. Over one year of follow-up, his blood pressure remained stable at 110–120/70–80 mmHg.

## 3 Discussion

The primary goal of perioperative management in PPGL is to normalize blood pressure and heart rate, ensure effective blood volume circulation, improve metabolic status, reduce catecholamine surges, and stabilize hemodynamics during surgery (4). The use of *α*-blockers alone restores blood volume in approximately 60% of PPGL patients. Therefore, guidelines recommend a high-sodium diet (5,000 mg daily), increased fluid intake, and rehydration therapy of 2,500∼3,000 ml M^2^ body surface area to reduce intraoperative blood pressure fluctuations (3, 5, 6).

In the described case, adequate perioperative management involved increasing the child's oral saline intake to approximately 45 ml/kg/d one week before surgery, which decreased hematocrit and increased weight. Intravenous blood volume replenishment before surgery stabilized hemodynamics, limiting blood pressure fluctuations to no more than 10 min beyond 30% of the baseline value.

Currently, there is no standardized protocol for preoperative blood volume supplementation in patients with pheochromocytoma. However, according to Roizen's criteria, adequate preoperative preparation includes: maintaining blood pressure below 160/90 mmHg, tolerable orthostatic hypotension above 80/45 mmHg, and indicators of blood volume recovery, such as decreased hematocrit, weight gain, limb warmth, and improved microcirculation. Additionally, hypermetabolic syndrome and abnormal glucose metabolism should be under control. Electrocardiographic (ECG) monitoring should show: no ST-T changes for at least one week, and no more than one ventricular extrasystole every 5 min (7, 8). In pediatric patients, blood pressure management must be individualized, as normal heart rate and blood pressure reference values vary with age.

During the first perioperative period, phentolamine mesylate, metoprolol, and amlodipine were used to manage blood pressure. Blood pressure decreased satisfactorily with increased saline intake to boost blood volume. Table 1 shows the dosages and side effects of commonly used preoperative medications for children with pheochromocytomas. Despite various preoperative hypotension protocols in the literature, large-scale prospective studies are not feasible due to the rarity and small sample size of the condition. Notably, using calcium antagonists alone for blood pressure control did not affect the incidence of perioperative complications.

**Table 1:** Commonly used medications for preoperative management of hypertension in PPGL.

Drug name	Mechanism	Dosage	Side effects
Phenoxybenzamine	Non-selective α1 and α2 blocker	0.2–0.25 mg/kg/d (max 10 mg/dose)	Orthostasis
			Nasal congestion
		Max, 2–4 mg/kg/d (60 mg/d)	Reflex tachycardia
Doxazosin	Selective α1 blocker	1–2 mg/d up to 2–4 m/d	Orthostasis
		Max, 4–16 mg/d, Q12H	Dizziness
Prazosin	Selective α1 blocker	0.05–0.1 mg/d, Q8H	Orthostasis
		Max, 0.5 mg/kg/d Q8H (20 mg/d)	Dizziness
Terazosin	Selective α1 blocker	1 mg/d up to 1–4 mg/d	Orthostasis
		Max, 20 mg/d	Dizziness
amlodipine	Dihydropyridine calcium channel blocker	0.1 mg/kg/d QD	Headache
			Edema
		Max, 0.5–0.6 mg/kg/d or 10 mg/d	Palpitations
Metyrosine	Inhibit tyrosine reduction	20 mg/kg/d, Q6H, or 125 mg/d, up to 60 mg/kg/d	Lethargy
			Extrapyramidal
		Max, 2,500 mg/d	Diarrhea
			Rarely crystalluria
Propranolol	Non-selective β1 and β2 blocker	1–2 mg/kg/d, Q6H-Q12H, up to 4 mg/kg/d Q6H-Q12H	Dizziness
		Max, 640 mg/d	Asthma flare-up
Atenolol	Selective β1 blocker	0.1–1 mg/kg/d, QD, up to 2 mg mg/kg/d QD	Dizziness
		Max, 100 mg/d	Fatigue
Metoprolol	Selective β1 blocker	1–2 mg/kg/d QD	Dizziness
		Max, 200 mg/d	
			Fatigue

The most common complication after tumor resection is hypotension (systolic blood pressure <80 mmHg, lasting 10 min), which can be managed with 500–1,000 ml colloid infusion and 3–9 mg ephedrine. If these measures are ineffective, epinephrine, norepinephrine, or methylprednisolone may be administered.

To date, cases of polyuria during preoperative preparation in pediatric patients with pheochromocytoma have not been reported. In this case, the child developed lower limb venous thrombosis, polyuria, multiple organ dysfunction, and infection, necessitating emergency surgery. During the operation, a left adrenal mass was identified but not removed due to the child's critical condition. In hindsight, performing a biopsy of the left adrenal mass might have been a more appropriate approach.

## 4 Conclusion

PPGL are rare endocrine tumors in children that necessitate careful perioperative management, including blood pressure reduction and heart rate control. The catecholamines secreted by PPGL cause vasoconstriction, leading to significant perioperative challenges such as hypotension and insufficient intravascular capacity, which require additional supplementation. Proper blood pressure control and adequate fluid management during preoperative preparation are essential to reduce the risk of both intraoperative hypertensive crises and postoperative hypotension. This case demonstrates that, with comprehensive evaluation and meticulous preoperative preparation by a multidisciplinary team, children with PPGL can safely navigate the perioperative period.

## Data Availability

The original contributions presented in the study are included in the article/Supplementary Material, further inquiries can be directed to the corresponding author.
